# Supplementary material for: Novel RNA viruses associated with Plasmodium vivax in human malaria and Leucocytozoon parasites in avian disease
Source: PLoS Pathog. 2019 Dec 30;15(12):e1008216. doi: 10.1371/journal.ppat.1008216 (PMC6953888; doi:10.1371/journal.ppat.1008216)
Supplement: S1 Table — PCR: PCR-based validation of Plasmodium species using species-specific primers: Pv—P. vivax; Pk—P. knowlesi; Pf—P. falciparum; pc—parasite counting (i.e. parasite density / μL = number of parasites counted x patient’s lab leukocyte result / 200 leukocytes counted). (DOCX) [file ppat.1008216.s001.docx]

**Table S1. Description of human blood samples used in this study.** PCR: PCR-based validation of *Plasmodium* species using species-specific primers: Pv - *P. vivax*; Pk - *P. knowlesi*; Pf - *P. falciparum*; pc - parasite counting (i.e. parasite density / μL = number of parasites counted x patient’s lab leukocyte result / 200 leukocytes counted).

| **Sample** | **Study** | **Date** | **Village** | **District** | **PCR** | **pc** |
| --- | --- | --- | --- | --- | --- | --- |
| **1** | QDM015 | Apr-14 | Talas | Kota Marudu | Pv | 4551 |
| **2** | MV001 | Jan-13 | Bambangan Ulu | Kota Marudu | Pv | 16968 |
| **3** | MV002 | Jan-13 | Sonsogon Magandai | Kota Marudu | Pv | 21952 |
| **4** | MV039 | Nov-14 | Sinar 9 Bombong | Kota Marudu | Pv | 3694 |
| **5** | MV036 | Oct-14 | Kalibambang | Kota Marudu | Pv | 10590 |
| **6** | MV038 | Nov-14 | Sinar 9 Bombong | Kota Marudu | Pv | 6027 |
| **10** | QDM014 | Apr-14 | Sonsogon Magandai | Kota Marudu | Pv | 4950 |
| **18** | QDM027 | May-14 | Baru Malalin | Belarun | Pk | 39565 |
| **19** | MK011 | Apr-13 | Togudon | Kota Belud | Pk | 3245 |
| **20** | MK072 | Aug-14 | Malangkap | Kota Marudu | Pk | 7177 |
| **21** | KK042 | Apr-13 | Balak-Balak | Kudat | Pk | 3363 |
| **22** | KK077 | Jan-14 | Kusilad Darat | Kudat | Pk | 5674 |
| **27** | QDM032 | May-14 | Hatob | Kota Marudu | Pk | 41882 |
| **28** | QEM782 | Jan-14 | Damaran | Kudat | Pf | 8199 |
| **31** | MF024 | Sep-14 | Matanggal | Kota Marudu | Pf | 8514 |
| **32** | MF028 | Oct-14 | Salimandut | Kota Marudu | Pf | 15207 |
| **33** | MF029 | Nov-14 | Gana | Kota Marudu | Pf | 6783 |
| **35** | QDM056 | Sep-14 | Sonsogon Magandai | Kota Marudu | Pf | 13977 |
| **38** | QECK037 | Oct-14 | Pantai Bahagia | Kudat | Negative | - |
| **39** | QECK039 | Oct-14 | Perpaduan | Kudat | Negative | - |
| **40** | QECK042 | Oct-14 | Lok Tohog | Pitas | Negative | - |
| **42** | QECK038 | Oct-14 | Batu 1/2 Jalan Atas | Kudat | Negative | - |
| **45** | QECK040 | Oct-14 | Penaitan | Kudat | Negative | - |
| **46** | QECK004 | Apr-13 | Taman Orkid Kudat | Kudat | Negative | - |
