# Supplementary material for: Novel RNA viruses associated with Plasmodium vivax in human malaria and Leucocytozoon parasites in avian disease
Source: PLoS Pathog. 2019 Dec 30;15(12):e1008216. doi: 10.1371/journal.ppat.1008216 (PMC6953888; doi:10.1371/journal.ppat.1008216)
Supplement: S2 Table — (DOCX) [file ppat.1008216.s002.docx]

**Table S2. BioProject and corresponding SRA accessions used in this study.**

| **Species** | **BioProject** | **No. libraries** |
| --- | --- | --- |
| ***P. chabaudi*** | PRJEB1284 | 60 |
|  | PRJEB1500 | 24 |
|  | PRJEB4572 | 84 |
|  | PRJEB4731 | 102 |
|  | PRJEB6241 | 78 |
|  | PRJEB6242 | 22 |
|  | **All** | **370** |
| ***P. cynomolgi*** | PRJNA356968 | 52 |
|  | **All** | **52** |
| ***P. falciparum*** | PRJEB19245 | 441 |
|  | PRJEB19644 | 60 |
|  | PRJEB21707 | 60 |
|  | PRJEB24218 | 128 |
|  | PRJEB25413 | 9 |
|  | PRJNA167166 | 3 |
|  | PRJNA308455 | 3 |
|  | PRJNA310220 | 12 |
|  | PRJNA315899 | 6 |
|  | PRJNA391508 | 16 |
|  | PRJNA401189 | 4 |
|  | **All** | **742** |
| ***P. knowlesi*** | PRJEB24220 | 69 |
|  | **All** | **69** |
| ***P. vivax*** | PRJEB15709 | 5 |
|  | PRJNA260605 | 19 |
|  | PRJNA279199 | 24 |
|  | PRJNA337969 | 55 |
|  | PRJNA376620 | 19 |
|  | PRJNA378759 | 4 |
|  | PRJNA422240 | 4 |
|  | PRJNA481383 | 52 |
|  | **All** | **182** |
| ***P. yoelli*** | PRJNA322665 | 16 |
|  | PRJNA394583 | 10 |
|  | **All** | **26** |
| ***P. berghei*** | PRJEB24219 | 52 |
|  | PRJNA286027 | 12 |
|  | PRJNA319543 | 124 |
|  | PRJNA374918 | 9 |
|  | PRJNA390648 | 3 |
|  | PRJNA391033 | 9 |
|  | PRJNA433164 | 32 |
|  | **All** | **241** |
|  | **TOTAL** | **1682** |
