# Supplementary material for: Novel RNA viruses associated with Plasmodium vivax in human malaria and Leucocytozoon parasites in avian disease
Source: PLoS Pathog. 2019 Dec 30;15(12):e1008216. doi: 10.1371/journal.ppat.1008216 (PMC6953888; doi:10.1371/journal.ppat.1008216)
Supplement: S3 Table — Libraries positive for MaRNAV-1 are shown in red and those used for phylogenetic analysis (MaRNAV-1-like read counts > 100) are shown in bold. Plasmodium-free libraries are in grey. Homo sapiens = human blood samples. Anopheles dirus = mosquito salivary gland dissection samples. Homo sapiens (ex vivo): Micropatterned cellular co-cultures. ND: Not determined. (DOCX) [file ppat.1008216.s003.docx]

**Table S3. *Plasmodium vivax* SRA libraries.** Libraries positive for MaRNAV-1 are shown in red and those used for phylogenetic analysis (MaRNAV-1-like read counts > 100) are shown in bold. *Plasmodium*-free libraries are in grey. *Homo sapiens* = human blood samples. *Anopheles dirus* = mosquito salivary gland dissection samples. *Homo sapiens (ex vivo)*: Micropatterned cellular co-cultures. ND: Not determined.

| **BioProject** | **SRA ID** | ***P. vivax* isolate** | **Locaton** | **Host** |
| --- | --- | --- | --- | --- |
| **PRJNA481383**  [[1]](https://paperpile.com/c/ncqrqF/Jogb) | SRR7554416 | *P. vivax* Thai_1 | Thailand | *A. dirus* |
|  | SRR7554417 | *P. vivax* Thai_1 | Thailand | *A. dirus* |
|  | SRR7554418 | *P. vivax* Thai_1 | Thailand | *A. dirus* |
|  | SRR7554419 | *P. vivax* Thai_1 | Thailand | *A. dirus* |
|  | SRR7554420 | *P. vivax* Thai_1 | Thailand | *A. dirus* |
|  | SRR7554421 | *P. vivax* Thai_1 | Thailand | *A. dirus* |
|  | SRR7554422 | *P. vivax* Thai_1 | Thailand | *A. dirus* |
|  | SRR7554423 | *P. vivax* Thai_1 | Thailand | *A. dirus* |
|  | SRR7554424 | *P. vivax* Thai_1 | Thailand | *A. dirus* |
|  | SRR7554425 | *P. vivax* Thai_1 | Thailand | *A. dirus* |
|  | SRR7554426 | *P. vivax* Thai_1 | Thailand | *A. dirus* |
|  | SRR7554427 | *P. vivax* Thai_1 | Thailand | *A. dirus* |
|  | SRR7554428 | *P. vivax* Thai_2 | Thailand | *A. dirus* |
|  | SRR7554429 | *P. vivax* Thai_2 | Thailand | *A. dirus* |
|  | SRR7554430 | *P. vivax* Thai_2 | Thailand | *A. dirus* |
|  | **SRR7554431** | ***P. vivax* Thai_2** | **Thailand** | ***A. dirus*** |
|  | SRR7554432 | *P. vivax* Thai_2 | Thailand | *A. dirus* |
|  | **SRR7554433** | ***P. vivax* Thai_2** | **Thailand** | ***A. dirus*** |
|  | SRR7554434 | *P. vivax* Thai_2 | Thailand | *A. dirus* |
|  | **SRR7554435** | ***P. vivax* Thai_2** | **Thailand** | ***A. dirus*** |
|  | **SRR7554436** | ***P. vivax* Thai_2** | **Thailand** | ***A. dirus*** |
|  | **SRR7554437** | ***P. vivax* Thai_2** | **Thailand** | ***A. dirus*** |
|  | **SRR7554438** | ***P. vivax* Thai_2** | **Thailand** | ***A. dirus*** |
|  | **SRR7554439** | ***P. vivax* Thai_2** | **Thailand** | ***A. dirus*** |
|  | SRR7554440 | *P. vivax* Thai_3 | Thailand | *A. dirus* |
|  | **SRR7554441** | ***P. vivax* Thai_3** | **Thailand** | ***A. dirus*** |
|  | SRR7554442 | *P. vivax* Thai_3 | Thailand | *A. dirus* |
|  | **SRR7554443** | ***P. vivax* Thai_3** | **Thailand** | ***A. dirus*** |
|  | SRR7554444 | *P. vivax* Thai_3 | Thailand | *A. dirus* |
|  | **SRR7554445** | ***P. vivax* Thai_3** | **Thailand** | ***A. dirus*** |
|  | SRR7554446 | *P. vivax* Thai_3 | Thailand | *A. dirus* |
|  | **SRR7554447** | ***P. vivax* Thai_3** | **Thailand** | ***A. dirus*** |
|  | SRR7554448 | *P. vivax* Thai_3 | Thailand | *A. dirus* |
|  | **SRR7554449** | ***P. vivax* Thai_3** | **Thailand** | ***A. dirus*** |
|  | SRR7554450 | *P. vivax* Thai_3 | Thailand | *A. dirus* |
|  | **SRR7554451** | ***P. vivax* Thai_3** | **Thailand** | ***A. dirus*** |
|  | SRR7554452 | *P. vivax* Thai_4 | Thailand | *A. dirus* |
|  | SRR7554453 | *P. vivax* Thai_4 | Thailand | *A. dirus* |
|  | SRR7554454 | *P. vivax* Thai_4 | Thailand | *A. dirus* |
|  | SRR7554455 | *P. vivax* Thai_4 | Thailand | *A. dirus* |
|  | SRR7554456 | *P. vivax* Thai_4 | Thailand | *A. dirus* |
|  | SRR7554457 | *P. vivax* Thai_4 | Thailand | *A. dirus* |
|  | SRR7554458 | *P. vivax* Thai_4 | Thailand | *A. dirus* |
|  | SRR7554459 | *P. vivax* Thai_4 | Thailand | *A. dirus* |
|  | **SRR7554460** | ***P. vivax* Thai_5** | **Thailand** | ***A. dirus*** |
|  | **SRR7554461** | ***P.vivax* Thai_5** | **Thailand** | ***A. dirus*** |
|  | **SRR7554462** | ***P.vivax* Thai_5** | **Thailand** | ***A. dirus*** |
|  | **SRR7554463** | ***P.vivax* Thai_5** | **Thailand** | ***A. dirus*** |
|  | *SRR7554464* | *Uninfected* | *ND* | *A. dirus* |
|  | *SRR7554465* | *Uninfected* | *ND* | *A. dirus* |
|  | *SRR7554466* | *Uninfected* | *ND* | *A. dirus* |
|  | *SRR7554467* | *Uninfected* | *ND* | *A. dirus* |
| **PRJNA422240**  [[2]](https://paperpile.com/c/ncqrqF/wKld) | SRR6371894 | *P. vivax*  (isolate VK210) #2 | Thailand | *Homo sapiens (ex vivo)* |
|  | SRR6371895 | *P. vivax*  (isolate VK210) #1 | Thailand | *Homo sapiens (ex vivo)* |
|  | SRR6371896 | *P. vivax*  (isolate VK210) #2 | Thailand | *Homo sapiens (ex vivo)* |
|  | SRR6371897 | *P. vivax*  (isolate VK210) #1 | Thailand | *Homo sapiens (ex vivo)* |
| **PRJNA378759**  [[3]](https://paperpile.com/c/ncqrqF/wKld+1s0E) | **SRR5646668** | **Sp_1** | **Colombia** | ***Anopheles albimanus*** |
|  | **SRR5646669** | **V_DJK_16** | **Cambodia** | ***Homo sapiens*** |
|  | SRR5646670 | V_DJK_10 | Cambodia | *Homo sapiens* |
|  | SRR5646671 | V_DJK_8 | Cambodia | *Homo sapiens* |
| **PRJNA376620**  [[4]](https://paperpile.com/c/ncqrqF/pk1H) | SRR5298172 | PvSPZ-Thai9 | Thailand | *A. dirus* |
|  | SRR5298173 | PvSPZ-Thai9 | Thailand | *A. dirus* |
|  | **SRR5298174** | **PvSPZ-Thai8** | **Thailand** | ***A. dirus*** |
|  | **SRR5298175** | **PvSPZ-Thai8** | **Thailand** | ***A. dirus*** |
|  | **SRR5298176** | **PvSPZ-Thai7** | **Thailand** | ***A. dirus*** |
|  | **SRR5298177** | **PvSPZ-Thai7** | **Thailand** | ***A. dirus*** |
|  | SRR5298178 | PvSPZ-Thai5 | Thailand | *A. dirus* |
|  | SRR5298179 | PvSPZ-Thai5 | Thailand | *A. dirus* |
|  | **SRR5298180** | **PvSPZ-Thai4** | **Thailand** | ***A. dirus*** |
|  | **SRR5298181** | **PvSPZ-Thai4** | **Thailand** | ***A. dirus*** |
|  | **SRR5298182** | **PvSPZ-Thai3** | **Thailand** | ***A. dirus*** |
|  | **SRR5298183** | **PvSPZ-Thai3** | **Thailand** | ***A. dirus*** |
|  | **SRR5298184** | **PvSPZ-Thai2** | **Thailand** | ***A. dirus*** |
|  | **SRR5298185** | **PvSPZ-Thai2** | **Thailand** | ***A. dirus*** |
|  | SRR5298186 | *P. falciparum (3D7)* | ND | *A. dirus* |
|  | SRR5298187 | *P. falciparum (3D7)* | ND | *A. dirus* |
|  | **SRR5298188** | **PvSPZ-Thai6** | **Thailand** | ***A. dirus*** |
|  | **SRR5298189** | **PvSPZ-Thai6** | **Thailand** | ***A. dirus*** |
|  | SRR5298190 | PvSPZ-Thai1 | Thailand | *A. dirus* |
| **PRJNA337969**  [[5]](https://paperpile.com/c/ncqrqF/ueY8)  *P. vivax challenge study* | *SRR4005681* | *Uninfected #10* | *-* | *Homo sapiens* |
|  | *SRR4005682* | *Uninfected #19* | *-* | *Homo sapiens* |
|  | *SRR4005683* | *Uninfected #5* | *-* | *Homo sapiens* |
|  | *SRR4005684* | *Uninfected #7* | *-* | *Homo sapiens* |
|  | *SRR4005685* | *Uninfected #3* | *-* | *Homo sapiens* |
|  | *SRR4005686* | *Uninfected #16* | *-* | *Homo sapiens* |
|  | *SRR4005687* | *Uninfected #1* | *-* | *Homo sapiens* |
|  | *SRR4005688* | *Uninfected #9* | *-* | *Homo sapiens* |
|  | *SRR4005689* | *Uninfected #11* | *-* | *Homo sapiens* |
|  | *SRR4005690* | *Uninfected #18* | *-* | *Homo sapiens* |
|  | *SRR4005691* | *Uninfected #12* | *-* | *Homo sapiens* |
|  | *SRR4005692* | *Uninfected #17* | *-* | *Homo sapiens* |
|  | *SRR4005693* | *Uninfected #13* | *-* | *Homo sapiens* |
|  | SRR4005694 | *P. vivax* - ND #4 | Colombia (Cali) | Homo sapiens |
|  | *SRR4005695* | *Uninfected #14* | *-* | *Homo sapiens* |
|  | *SRR4005696* | *Uninfected #6* | *-* | *Homo sapiens* |
|  | *SRR4005697* | *Uninfected #2* | *-* | *Homo sapiens* |
|  | *SRR4005698* | *Uninfected #8* | *-* | *Homo sapiens* |
|  | *SRR4005699* | *Uninfected #15* | *-* | *Homo sapiens* |
|  | *SRR4005700* | *Uninfected #10* | *-* | *Homo sapiens* |
|  | SRR4005701 | *P. vivax* *-* ND #19 | Colombia (Cali) | *Homo sapiens* |
|  | *SRR4005702* | *Uninfected #3* | *-* | *Homo sapiens* |
|  | *SRR4005703* | *Uninfected #16* | *-* | *Homo sapiens* |
|  | *SRR4005704* | *Uninfected #1* | *-* | *Homo sapiens* |
|  | *SRR4005705* | *Uninfected #9* | *-* | *Homo sapiens* |
|  | SRR4005706 | *P. vivax* *-* ND #11 | Colombia (Cali) | *Homo sapiens* |
|  | SRR4005707 | *P. vivax -* ND #12 | Colombia (Cali) | *Homo sapiens* |
|  | SRR4005708 | *P. vivax* *-* ND #17 | Colombia (Cali) | *Homo sapiens* |
|  | SRR4005709 | *P. vivax* - ND #13 | Colombia (Cali) | *Homo sapiens* |
|  | SRR4005710 | *P. vivax -* ND #4 | Colombia (Cali) | *Homo sapiens* |
|  | SRR4005711 | *P. vivax* - ND #14 | Colombia (Cali) | *Homo sapiens* |
|  | *SRR4005712* | *Uninfected #6* | *-* | *Homo sapiens* |
|  | *SRR4005713* | *Uninfected #2* | *-* | *Homo sapiens* |
|  | *SRR4005714* | *Uninfected #8* | *-* | *Homo sapiens* |
|  | *SRR4005715* | *Uninfected #15* | *-* | *Homo sapiens* |
|  | *SRR4005716* | *Uninfected #20* | *-* | *Homo sapiens* |
|  | *SRR4005717* | *Uninfected #10* | *-* | *Homo sapiens* |
|  | *SRR4005718* | *Uninfected #5* | *-* | *Homo sapiens* |
|  | *SRR4005719* | *Uninfected #7* | *-* | *Homo sapiens* |
|  | *SRR4005720* | *Uninfected #3* | *-* | *Homo sapiens* |
|  | *SRR4005721* | *Uninfected #16* | *-* | *Homo sapiens* |
|  | *SRR4005722* | *Uninfected #1* | *-* | *Homo sapiens* |
|  | *SRR4005723* | *Uninfected #9* | *-* | *Homo sapiens* |
|  | *SRR4005724* | *Uninfected #11* | *-* | *Homo sapiens* |
|  | *SRR4005725* | *Uninfected #18* | *-* | *Homo sapiens* |
|  | *SRR4005726* | *Uninfected #12* | *-* | *Homo sapiens* |
|  | *SRR4005727* | *Uninfected #17* | *-* | *Homo sapiens* |
|  | *SRR4005728* | *Uninfected #13* | *-* | *Homo sapiens* |
|  | SRR4005729 | *P. vivax* - ND #4 | Colombia (Cali) | *Homo sapiens* |
|  | *SRR4005730* | *Uninfected #14* | *-* | *Homo sapiens* |
|  | *SRR4005731* | *Uninfected #6* | *-* | *Homo sapiens* |
|  | *SRR4005732* | *Uninfected #2* | *-* | *Homo sapiens* |
|  | *SRR4005733* | *Uninfected #8* | *-* | *Homo sapiens* |
|  | *SRR4005734* | *Uninfected #15* | *-* | *Homo sapiens* |
|  | *SRR4005735* | *Uninfected #20* | *-* | *Homo sapiens* |
| **PRJNA279199**  [[6]](https://paperpile.com/c/ncqrqF/iUkt)  *P. vivax challenge study* | SRR1925781 | *P. vivax* - ND #1 | Colombia (Buenaventura) | *Homo sapiens* |
|  | *SRR1925782* | *Uninfected #2* | *-* | *Homo sapiens* |
|  | SRR1925783 | *P. vivax* - ND #3 | Colombia (Buenaventura) | *Homo sapiens* |
|  | *SRR1925784* | *Uninfected #4* | *-* | *Homo sapiens* |
|  | SRR1925785 | *P. vivax* - ND #5 | Colombia (Buenaventura) | *Homo sapiens* |
|  | *SRR1925786* | *Uninfected #1* | *-* | *Homo sapiens* |
|  | SRR1925787 | *P. vivax* - ND #6 | Colombia (Buenaventura) | *Homo sapiens* |
|  | SRR1925788 | *P. vivax* - ND #7 | Colombia (Buenaventura) | *Homo sapiens* |
|  | *SRR1925789* | *Uninfected #9* | *-* | *Homo sapiens* |
|  | SRR1925790 | *P. vivax* - ND #9 | Colombia (Buenaventura) | *Homo sapiens* |
|  | SRR1925791 | *P. vivax* - ND #10 | Colombia (Buenaventura) | *Homo sapiens* |
|  | *SRR1925792* | *Uninfected #3* | *-* | *Homo sapiens* |
|  | *SRR1925793* | *Uninfected #11* | *-* | *Homo sapiens* |
|  | *SRR1925794* | *Uninfected #6* | *-* | *Homo sapiens* |
|  | SRR1925795 | *P. vivax* - ND #4 | Colombia (Buenaventura) | *Homo sapiens* |
|  | *SRR1925796* | *Uninfected #12* | *-* | *Homo sapiens* |
|  | SRR1925797 | *P. vivax* - ND #12 | Colombia (Buenaventura) | *Homo sapiens* |
|  | SRR1925798 | *P. vivax* - ND #2 | Colombia (Buenaventura) | *Homo sapiens* |
|  | SRR1925799 | *P. vivax* - ND #8 | Colombia (Buenaventura) | *Homo sapiens* |
|  | *SRR1925800* | *Uninfected #9* | *-* | *Homo sapiens* |
|  | *SRR1925801* | *Uninfected #5* | *-* | *Homo sapiens* |
|  | *SRR1925802* | *Uninfected #7* | *-* | *Homo sapiens* |
|  | SRR1925803 | *P. vivax* - ND #11 | Colombia (Buenaventura) | *Homo sapiens* |
|  | *SRR1925804* | *Uninfected #10* | *-* | *Homo sapiens* |
| **PRJNA260605**  [[7]](https://paperpile.com/c/ncqrqF/q3lU) | SRR1571697 | *P. vivax* - SMRU #1 | Thailand | *Ex vivo* culture |
|  | SRR1571698 | *P. vivax* - SMRU #1 | Thailand | *Ex vivo* culture |
|  | SRR1571699 | *P. vivax* - SMRU #1 | Thailand | *Ex vivo* culture |
|  | SRR1571700 | *P. vivax* - SMRU #1 | Thailand | *Ex vivo* culture |
|  | SRR1571701 | *P. vivax* - SMRU #1 | Thailand | *Ex vivo* culture |
|  | SRR1571702 | *P. vivax* - SMRU #1 | Thailand | *Ex vivo* culture |
|  | SRR1571703 | *P. vivax* - SMRU #1 | Thailand | *Ex vivo* culture |
|  | SRR1571704 | *P. vivax* - SMRU #2 | Thailand | *Ex vivo* culture |
|  | SRR1571705 | *P. vivax* - SMRU #2 | Thailand | *Ex vivo* culture |
|  | SRR1571706 | *P. vivax* - SMRU #2 | Thailand | *Ex vivo* culture |
|  | SRR1571707 | *P. vivax* - SMRU #2 | Thailand | *Ex vivo* culture |
|  | SRR1571708 | *P. vivax* - SMRU #2 | Thailand | *Ex vivo* culture |
|  | SRR1571709 | *P. vivax* - SMRU #2 | Thailand | *Ex vivo* culture |
|  | SRR1571710 | *P. vivax* - SMRU #2 | Thailand | *Ex vivo* culture |
|  | SRR1571711 | *P. vivax* - SMRU #2 | Thailand | *Ex vivo* culture |
|  | SRR1571712 | *P. vivax* - Mixed | Thailand | *Ex vivo* culture |
|  | SRR1571713 | *P. vivax* - Mixed | Thailand | *Ex vivo* culture |
|  | SRR1571714 | P*. vivax* - Mixed | Thailand | *Ex vivo* culture |
|  | SRR1571715 | *P. vivax* - Mixed | Thailand | *Ex vivo* culture |
| **PRJEB15709** | **ERR1717084** | ***P. vivax*** | **ND** | ***Homo sapiens*** |
|  | **ERR1717085** | ***P. vivax*** | **ND** | ***Homo sapiens*** |
|  | **ERR1717086** | ***P. vivax*** | **ND** | ***Homo sapiens*** |
|  | ERR1717087 | *P. vivax* | ND | *Homo sapiens* |
|  | ERR1717452 | *P. vivax* | ND | *Homo sapiens* |
