# Supplementary material for: Novel RNA viruses associated with Plasmodium vivax in human malaria and Leucocytozoon parasites in avian disease
Source: PLoS Pathog. 2019 Dec 30;15(12):e1008216. doi: 10.1371/journal.ppat.1008216 (PMC6953888; doi:10.1371/journal.ppat.1008216)
Supplement: S4 Table — (DOCX) [file ppat.1008216.s004.docx]

Table S4. **BioProject and corresponding SRA accessions of *P. vivax*-free Anopheles species.**

| Anopheles species | Run | BioProject |
| --- | --- | --- |
| *Anopheles stephensi* | SRR8501109 | PRJNA517570 |
|  | SRR8501108 | PRJNA517570 |
|  | SRR3195175 | PRJNA313468 |
|  | SRR1851022 | PRJNA277477 |
|  | SRR1851027 | PRJNA277477 |
|  | SRR1448248 | PRJNA253267 |
|  | SRR1447827 | PRJNA253267 |
|  | SRR1448245 | PRJNA253267 |
|  | SRR1462338 | PRJNA253267 |
|  | SRR1755740 | PRJNA238706 |
|  | SRR1755745 | PRJNA238705 |
|  | SRR1755787 | PRJNA238704 |
|  | SRR1755915 | PRJNA238703 |
|  | SRR606157 | PRJNA173646 |
|  | SRR606152 | PRJNA173646 |
|  | SRR606158 | PRJNA173646 |
|  | ERR1095203 | PRJEB11516 |
|  | ERR1095202 | PRJEB11516 |
|  | ERR1095201 | PRJEB11516 |
|  | ERR1095200 | PRJEB11516 |
|  | SRR606151 | PRJNA173646 |
| *Anopheles dirus* | SRR826831 | PRJNA196855 |
|  | SRR908280 | PRJNA208524 |
|  | SRR6235142 | PRJNA416343 |
|  | SRR6235144 | PRJNA416343 |
| *Anopheles sinensis* | SRR1143271 | PRJNA236162 |
|  | SRR5313184 | PRJNA377961 |
|  | SRR5313183 | PRJNA377961 |
|  | SRR5313185 | PRJNA377961 |
| *Anopheles minimus* | SRR4104246 | PRJNA340355 |
|  | SRR4104220 | PRJNA340355 |
|  | SRR4104278 | PRJNA340355 |
|  | SRR4104223 | PRJNA340355 |
|  | SRR826833 | PRJNA196858 |
| *Anopheles sinensis* | SRR3589929 | PRJNA321832 |
|  | SRR851144 | PRJNA196809 |
| *Anopheles culicifacies* | SRR6260436 | PRJNA414162 |
|  | SRR1959072 | PRJNA275688 |
| *Anopheles farauti* | SRR606156 | PRJNA173643 |
|  | SRR606161 | PRJNA173643 |
|  | SRR606162 | PRJNA173643 |
|  | SRR606155 | PRJNA173643 |
