# Supplementary material for: Novel RNA viruses associated with Plasmodium vivax in human malaria and Leucocytozoon parasites in avian disease
Source: PLoS Pathog. 2019 Dec 30;15(12):e1008216. doi: 10.1371/journal.ppat.1008216 (PMC6953888; doi:10.1371/journal.ppat.1008216)
Supplement: S5 Table — Presence, absence or uncertainty of Leucocytozoon detection from pathology reports are highlighted in green, red and orange, respectively. Positive or negative PCR targeting either the Leucocytozoon parasite, the RdRP-like segment and the unknown second segment of MaRNAV-2 are highlighted in green and red, respectively. (DOCX) [file ppat.1008216.s005.docx]

**Table S5. Bird sample analysis summary table.** Presence, absence or uncertainty of *Leucocytozoon* detection from pathology reports are highlighted in green, red and orange, respectively. Positive or negative PCR targeting either the *Leucocytozoon* parasite, the RdRP-like segment and the unknown second segment of MaRNAV-2 are highlighted in green and red, respectively.
