# Supplementary material for: Novel RNA viruses associated with Plasmodium vivax in human malaria and Leucocytozoon parasites in avian disease
Source: PLoS Pathog. 2019 Dec 30;15(12):e1008216. doi: 10.1371/journal.ppat.1008216 (PMC6953888; doi:10.1371/journal.ppat.1008216)
Supplement: S6 Table — (DOCX) [file ppat.1008216.s006.docx]

**Table S6.** Primers used in this study.

| **Name** | **Sequence (5’ – 3’)** | **Application** |
| --- | --- | --- |
| *P. vivax* _Fw | CGGCTTGGAAGTCCTTGT | *P. vivax and P. falciparum* validation in blood samples.  From [8] |
| *P. falciparum*_Fw | AACAGACGGGTAGTCATGATTGAG |  |
| *Plasmodium*_Rev | GTATCTGATCGTCTTCACTCCC |  |
| PkF1140 | GATTCATCTATTAAAAATTTGCTTC | *P. knowlesi* validation in blood samples From [9] |
| PkR1150 | TCTTTTTCTCCGGAGATTAGAACTC |  |
| PkF1160 | GATGCCTCCGCGTATCGAC |  |
| rPLU3 | TTTTTATAAGGATAACTACGGAAAAGCTGT | *P. knowlesi* validation in blood samples From [10] |
| rPLU4 | TACCCGTCATAGCCATGTTAGGCCAATACC |  |
| Human_RPS18_Fw | ATGCAGAATCCACGCCAGTA | Human mRNA Detection |
| Human_RPS18_Rev | CCAGACCATTGGCTAGGACC |  |
| *Plasmodium*_LDH-P_Fw | ﻿GCTTTTCCTTGGGGCATGTT | *Plasmodium* mRNA detection |
| *Plasmodium*_LDH-P_Rev | GGTATGATCGGAGGAGTGATGG |  |
| MARNAV-1_Fw_5 | GACTCGTCACCTTGTGAGGC | MARNAV-1 detection (segment I) |
| MARNAV-1_Rev_5 | TGGCATCCACTTCAAGCAGG |  |
| MARNAV-1_Fw1 | TTGTCGGTGGAACTCCTTCG | MARNAV-1 full length amplification  (segment I) |
| MARNAV-1_Rev4 | ACATCCAAGCAACACACCCT |  |
| MARNAV-1_Fw3 | GCCTCACAAGGTGACGAGTC | MaRNAV-1 sequencing (segment I) |
| MARNAV-1_Fw4 | CCTAAGGCGTTCCCTCCTTC |  |
| MARNAV-1_Rev1 | CCTGCTTGAAGTGGATGCCA |  |
| MARNAV-1_Rev2 | TCCAGTTTTCATCGGCAGGA |  |
| MARNAV-1_Rev3 | AAGGCGTCACACCTCAGTAG |  |
| MARNAV-1_Fw3 | GCCTCACAAGGTGACGAGTC |  |
| Pv_1_unknown_contig Fw1 | GGCGTACTCGTTGCTTTTGT | MARNAV-1 detection, full-length amplification and sequencing (segment II) |
| Pv_1_unknown_contig Rev1 | GTTGGAAGAGCTGCAGAGGT |  |
| Pv_1_unknown_contig Rev2 | AATCCTGTCGCGGACACAAT |  |
| BW_Narnalike_Fw1 | CTGAAATTGATAARGAYGAAACTCC | Bird samples MARNAV-2 detection (segment I) |
| BW_Narnalike_Rev1 | CGTGGCATCCTTYAAATCTGATG |  |
| Haem cytB_AE986-F | AGTGGATGGTGYTTYAGATAYTTAC | Bird samples hematozoa cytB detection From [11] |
| Haem cytB_AE066-IR | GCTTGGGAGCTGTAATCATAAT |  |
| BW.Narna.Novel.3F | TCCATAAATGATGGGAGTAATCGC | Bird samples MARNAV-2 detection (segment II) |
| BW.Narna.Novel.2R | GATCTTGTATATACATAGATCCAATACAG |  |
