# Supplementary material for: Novel RNA viruses associated with Plasmodium vivax in human malaria and Leucocytozoon parasites in avian disease
Source: PLoS Pathog. 2019 Dec 30;15(12):e1008216. doi: 10.1371/journal.ppat.1008216 (PMC6953888; doi:10.1371/journal.ppat.1008216)
Supplement: S7 Table — (DOCX) [file ppat.1008216.s007.docx]

**Table S7.** Quality of RNA extraction and RNA-seq data sets obtained.

| ***Plasmodium* species** | **Sample ID** | **RNA extraction set** | **Total RNA quality (RIN)** | **RNA-Seq depth**  **(read number)** |
| --- | --- | --- | --- | --- |
| ***P. vivax*** | 1 | A | 8.1 | 17,532,644 |
|  | 2 | A | 7.4 |  |
|  | 3 | B | 8 |  |
|  | 4 | B | 6.2 |  |
|  | 5 | C | 6.2 |  |
|  | 6 | C | 6.9 |  |
|  | 10 | F | 6.3 |  |
| ***P. knowlesi*** | 18 | A | 8.2 | 17,082,864 |
|  | 19 | A | 8.2 |  |
|  | 20 | B | 7.4 |  |
|  | 21 | B | 7.3 |  |
|  | 22 | C | 6.5 |  |
|  | 27 | F | 7 |  |
| ***P. falciparum*** | 28 | A | 6.9 | 16,666,960 |
|  | 31 | C | 7.9 |  |
|  | 32 | C | 6.6 |  |
|  | 33 | D | 8.7 |  |
|  | 35 | E | 7.4 |  |
| **Uninfected** | 38 | A | 6.1 | 16,314,125 |
|  | 39 | B | 7.1 |  |
|  | 40 | B | 6.2 |  |
|  | 42 | C | 7.1 |  |
|  | 45 | E | 5.9 |  |
|  | 46 | F | 7 |  |
