# Supplementary material for: Novel RNA viruses associated with Plasmodium vivax in human malaria and Leucocytozoon parasites in avian disease
Source: PLoS Pathog. 2019 Dec 30;15(12):e1008216. doi: 10.1371/journal.ppat.1008216 (PMC6953888; doi:10.1371/journal.ppat.1008216)
Supplement: S8 Table — (DOCX) [file ppat.1008216.s008.docx]

**Table S8. List of databases and software used for rRNA and host read depletion**

| **Depletion** | **Reference/accession** | **Reference link** | **Software** | **Software reference** |
| --- | --- | --- | --- | --- |
| **rRNA** | Silva-arc-16s-id95 | [12] | SortmeRNA | [13] |
|  | Silva-arc-23s-id98 |  |  |  |
|  | Silva-bac-16s-id90 |  |  |  |
|  | Silva-bac-23s-id98 |  |  |  |
|  | Silva-euk-18s-id95 |  |  |  |
|  | Silva-euk-28s-id98 |  |  |  |
| **Short non-coding rRNA** | Rfam-5.8s-database-id98 | [14, 15] |  |  |
|  | Rfam-5s-database-id98 |  |  |  |
| **Human** | Refseq \| GCF_000001405.38 |  | Bowtie2 | [16] |
| ***P. vivax*** | Refseq \| GCF_000002415.2 |  |  |  |
| ***P. knowlesi*** | Refseq \| GCF_000006355.1 |  |  |  |
| ***P. falciparum*** | Refseq \| GCF_000002765.4 |  |  |  |
