# Supplementary figures and images for: Novel RNA viruses associated with Plasmodium vivax in human malaria and Leucocytozoon parasites in avian disease
Source: PLoS Pathog. 2019 Dec 30;15(12):e1008216. doi: 10.1371/journal.ppat.1008216 (PMC6953888; doi:10.1371/journal.ppat.1008216)

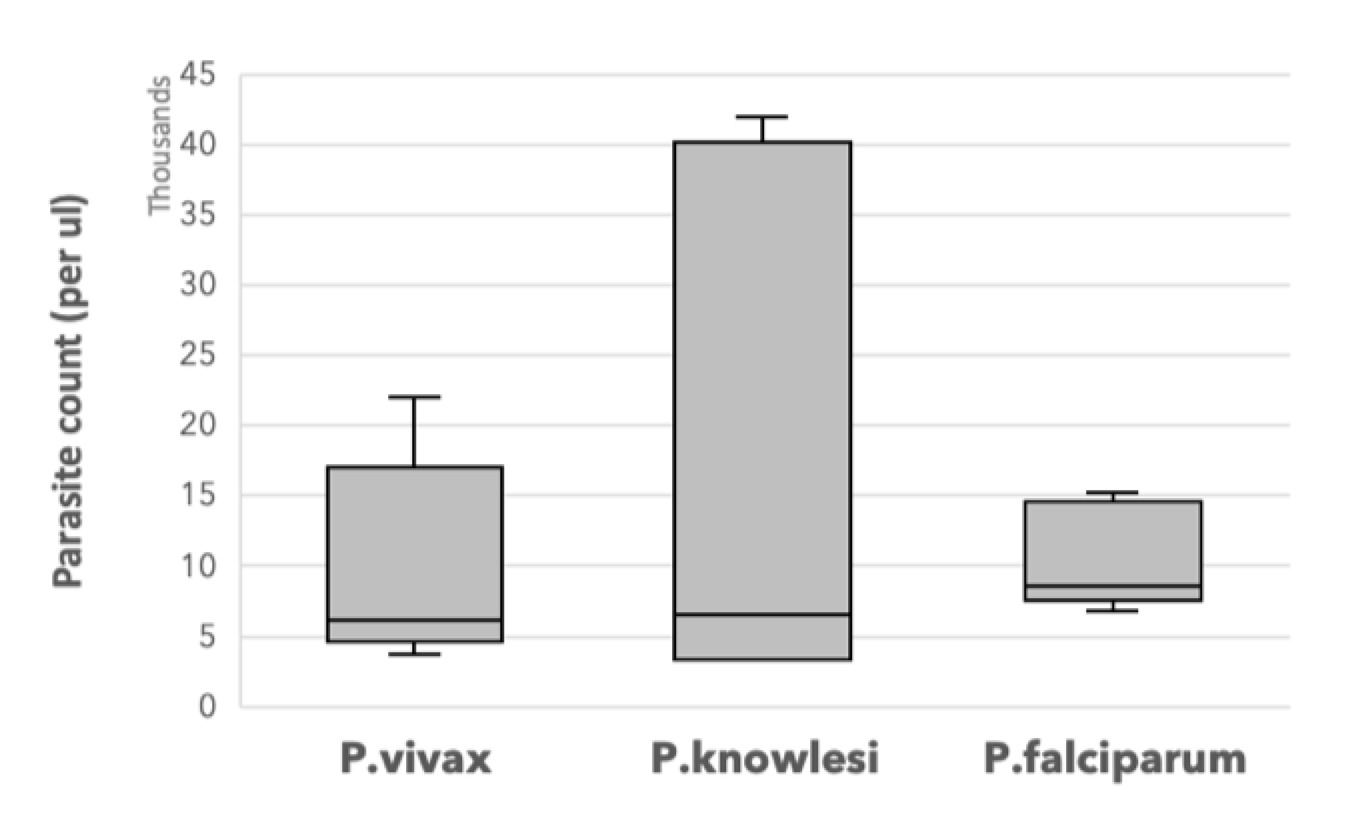

Supplement: S1 Fig — Parasite counts are expressed as the number of parasites per μl of blood. (TIF) [file ppat.1008216.s009.tif]

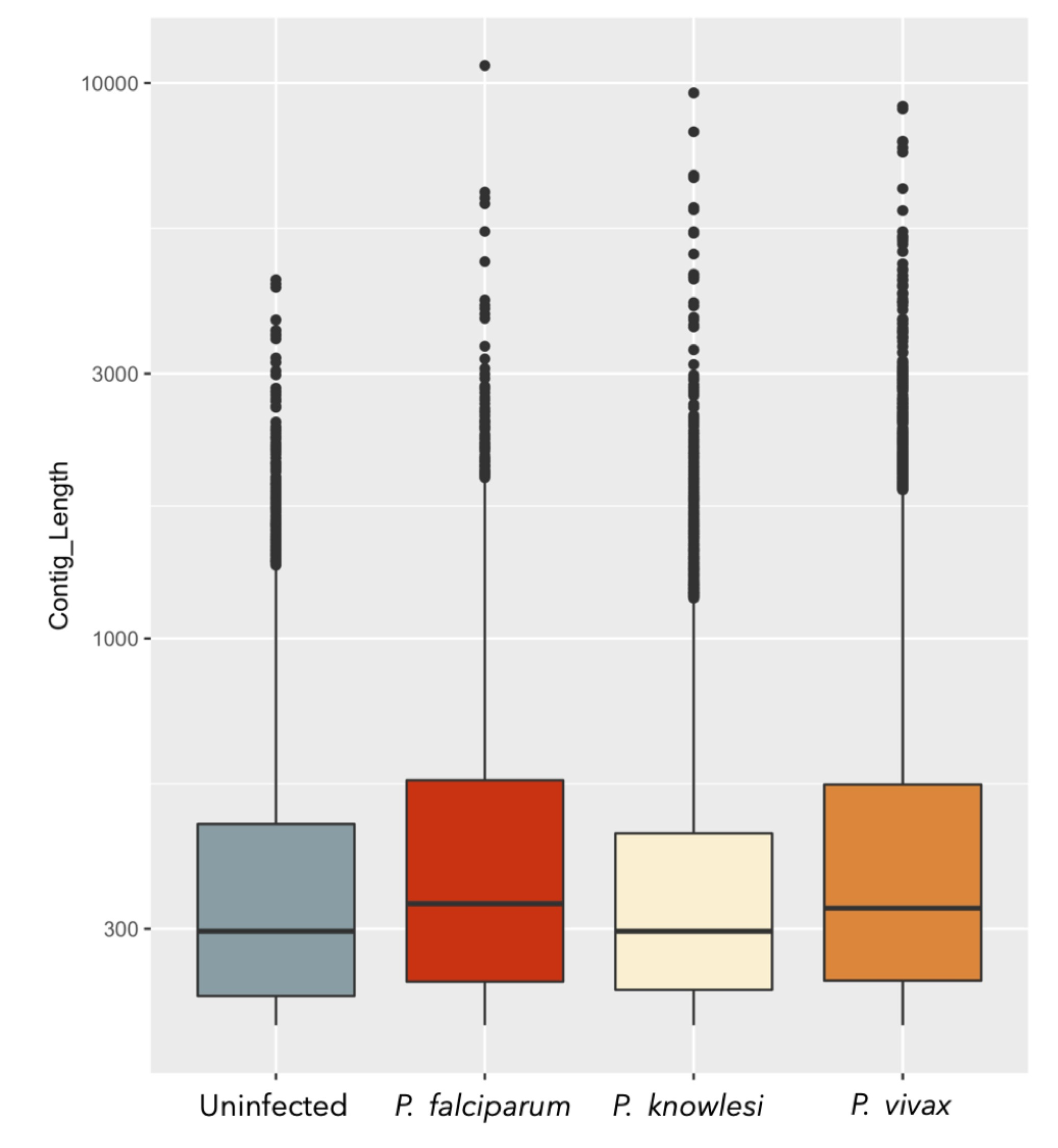

Supplement: S2 Fig — Contig length and count obtained after performing Trinity assembly of libraries depleted in rRNA, human and Plasmodium reads. (TIF) [file ppat.1008216.s010.tif]

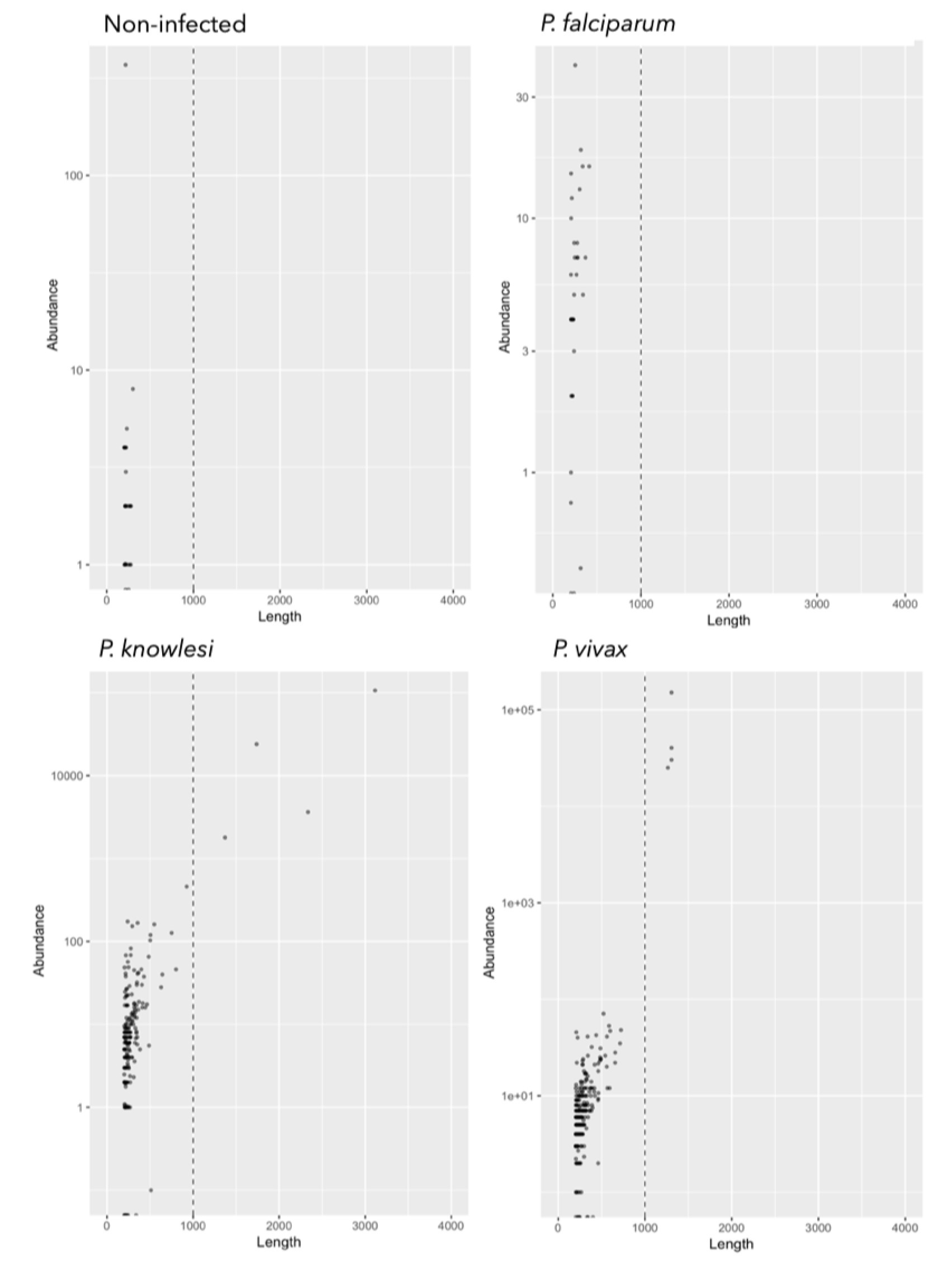

Supplement: S3 Fig — An arbitrary cut-off of 1000 nt was used to identify candidate RNA viruses. Abundance is expressed using the expected count value provided by the RSEM analysis. (TIF) [file ppat.1008216.s011.tif]

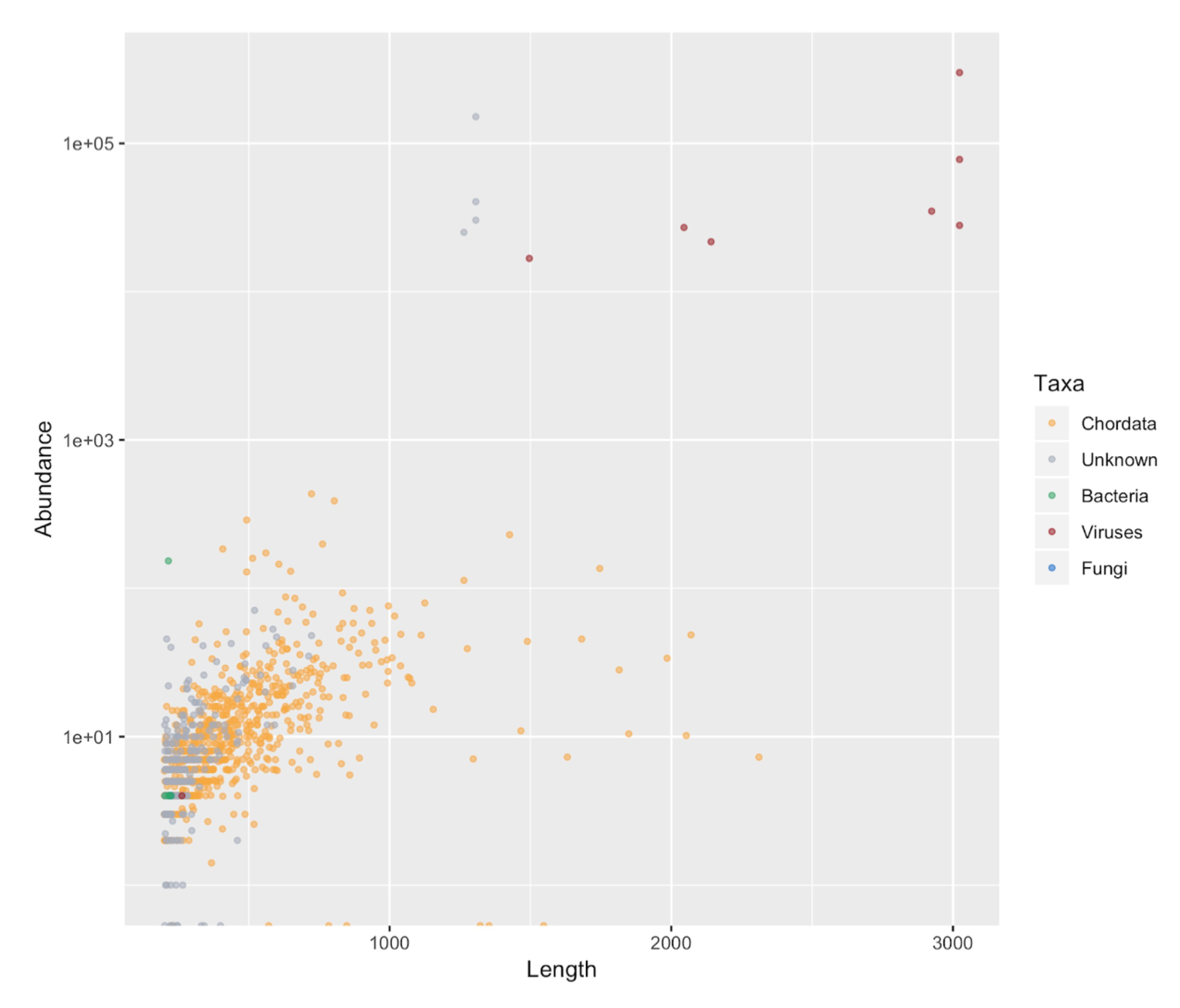

Supplement: S4 Fig — Abundance is expressed using the expected count value provided by the RSEM analysis. (TIF) [file ppat.1008216.s012.tif]

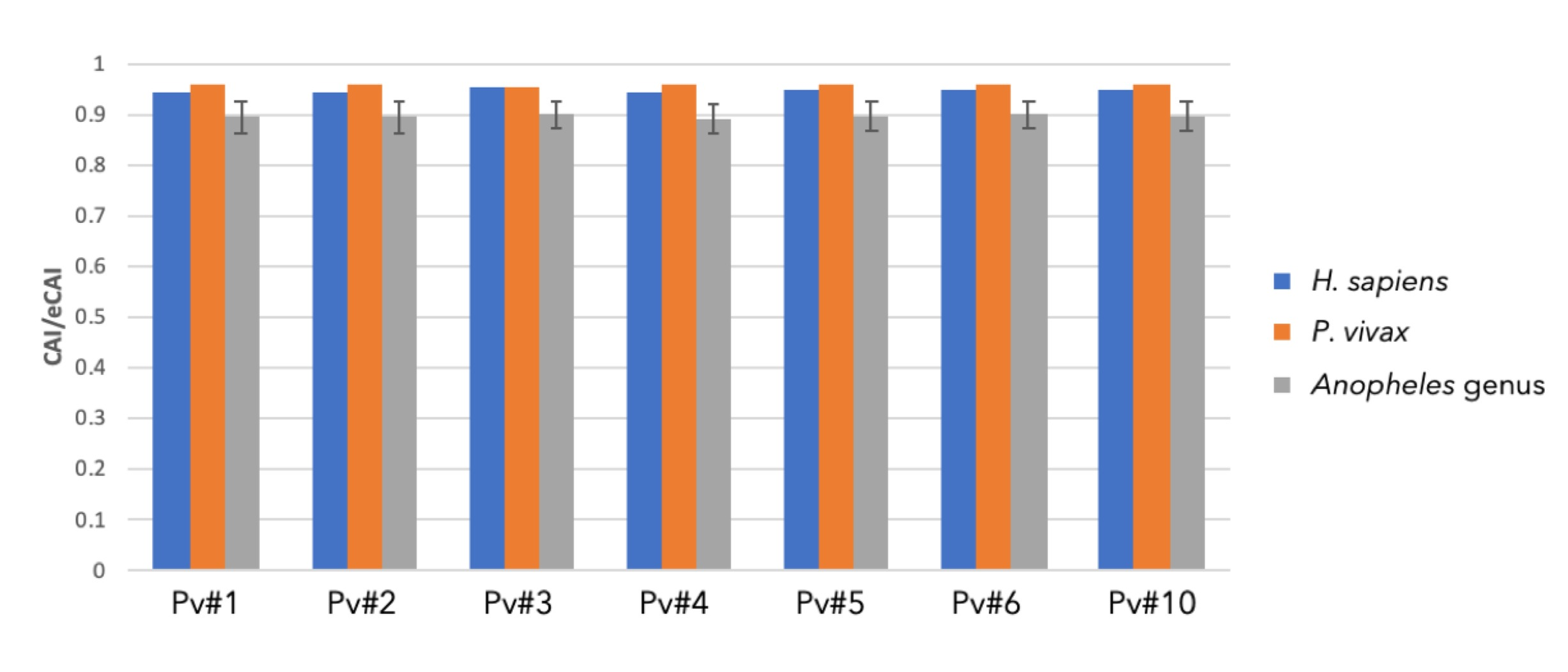

Supplement: S5 Fig — (TIF) [file ppat.1008216.s013.tif]

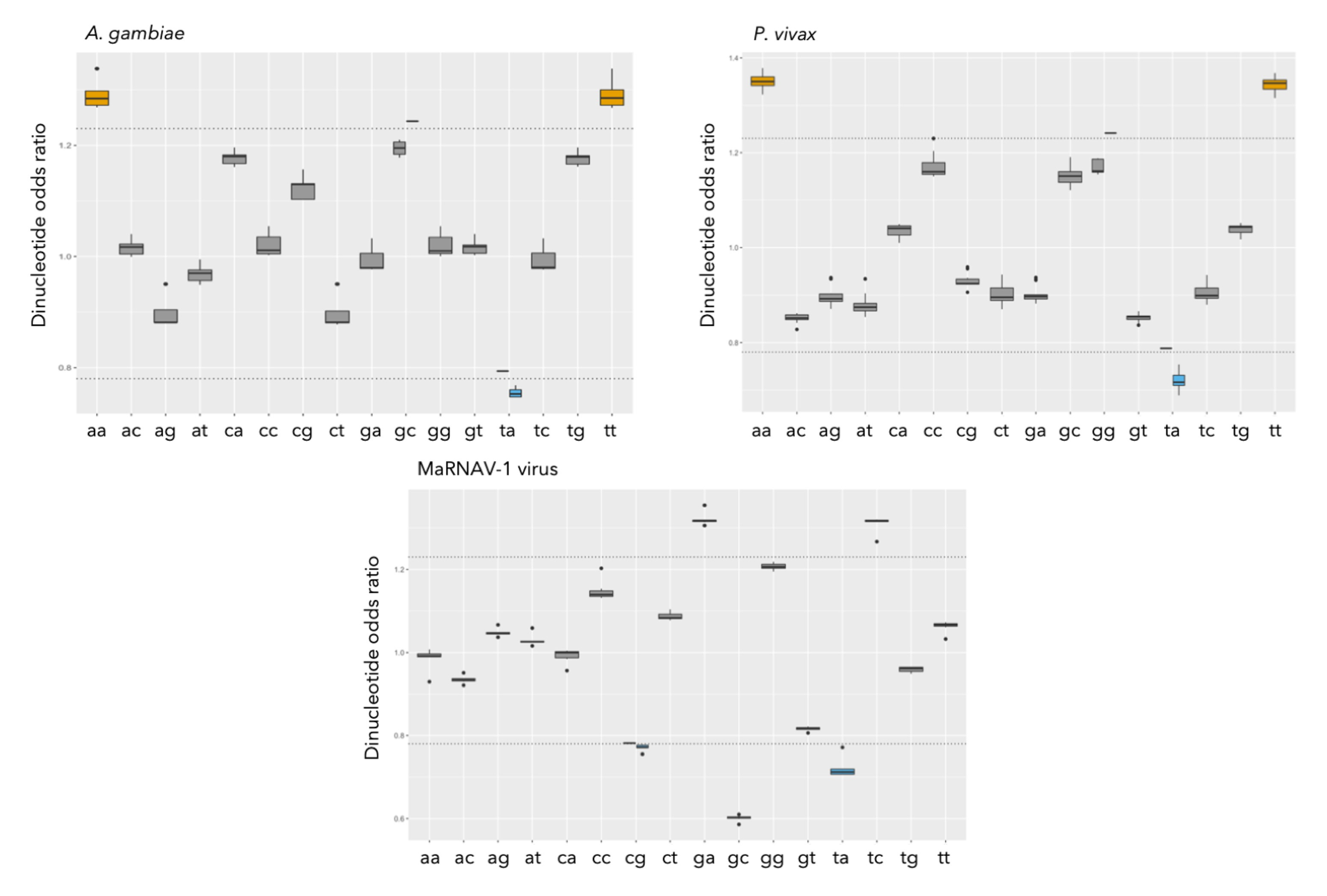

Supplement: S6 Fig — (TIF) [file ppat.1008216.s014.tif]

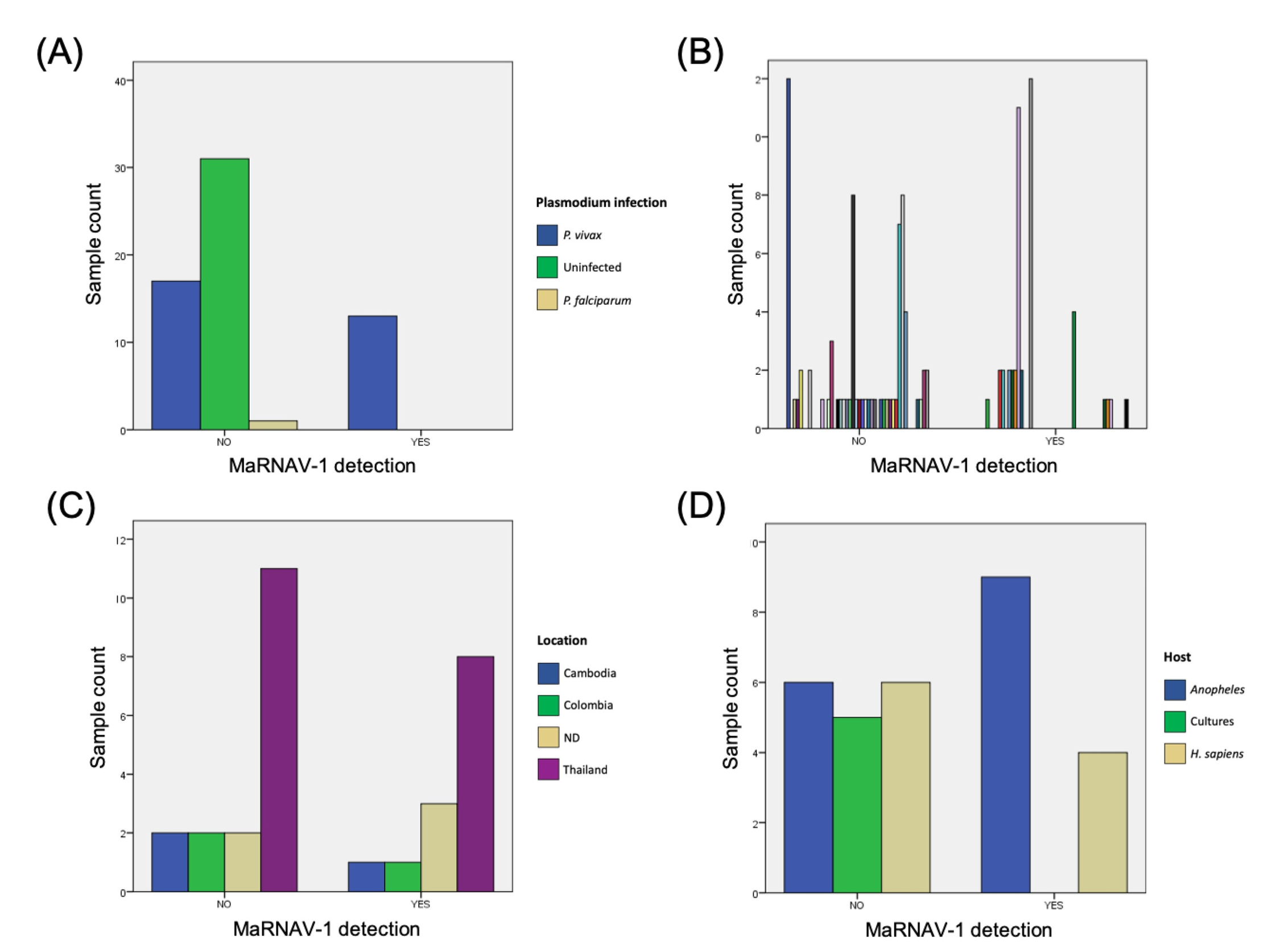

Supplement: S7 Fig — (A) Plasmodium infection association test. (B) Biological replicates association test. Replicates corresponding to the same biological sample are grouped by colour. (C) Sample location association test. (D) Host association test. (TIF) [file ppat.1008216.s015.tif]

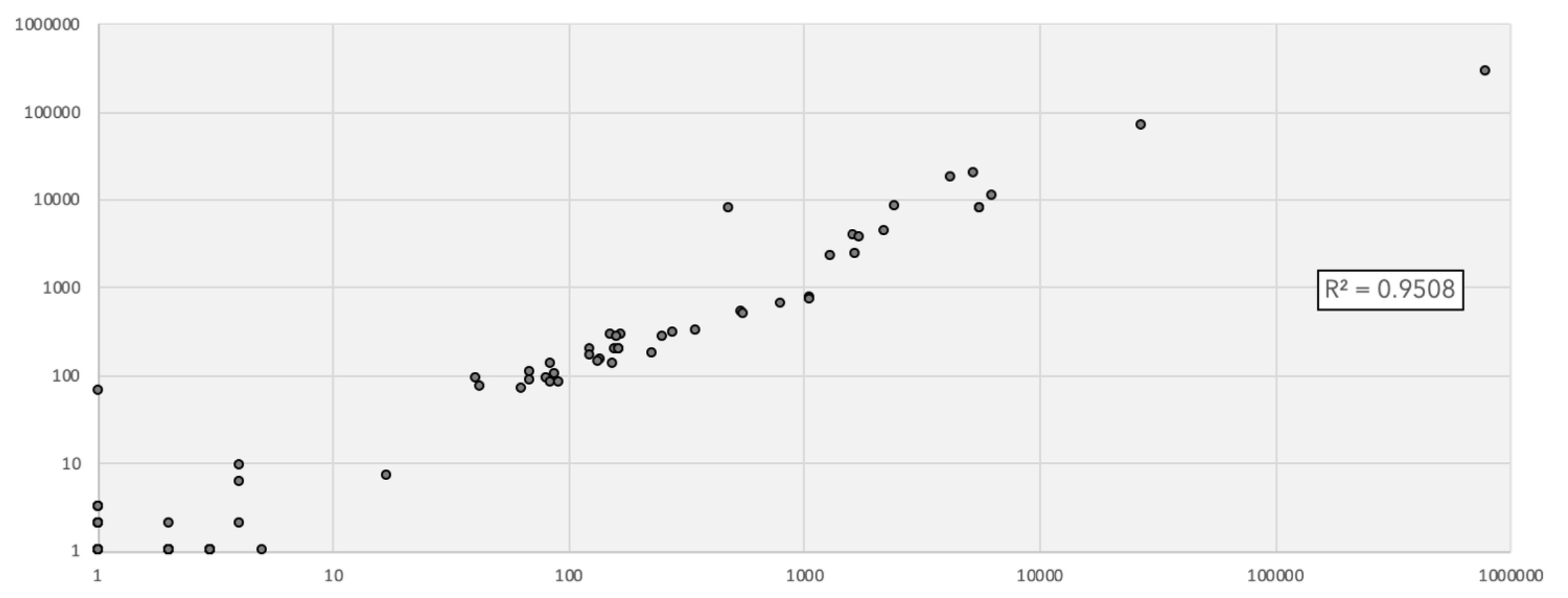

Supplement: S8 Fig — The R-squared value is indicated on the right. (TIF) [file ppat.1008216.s016.tif]

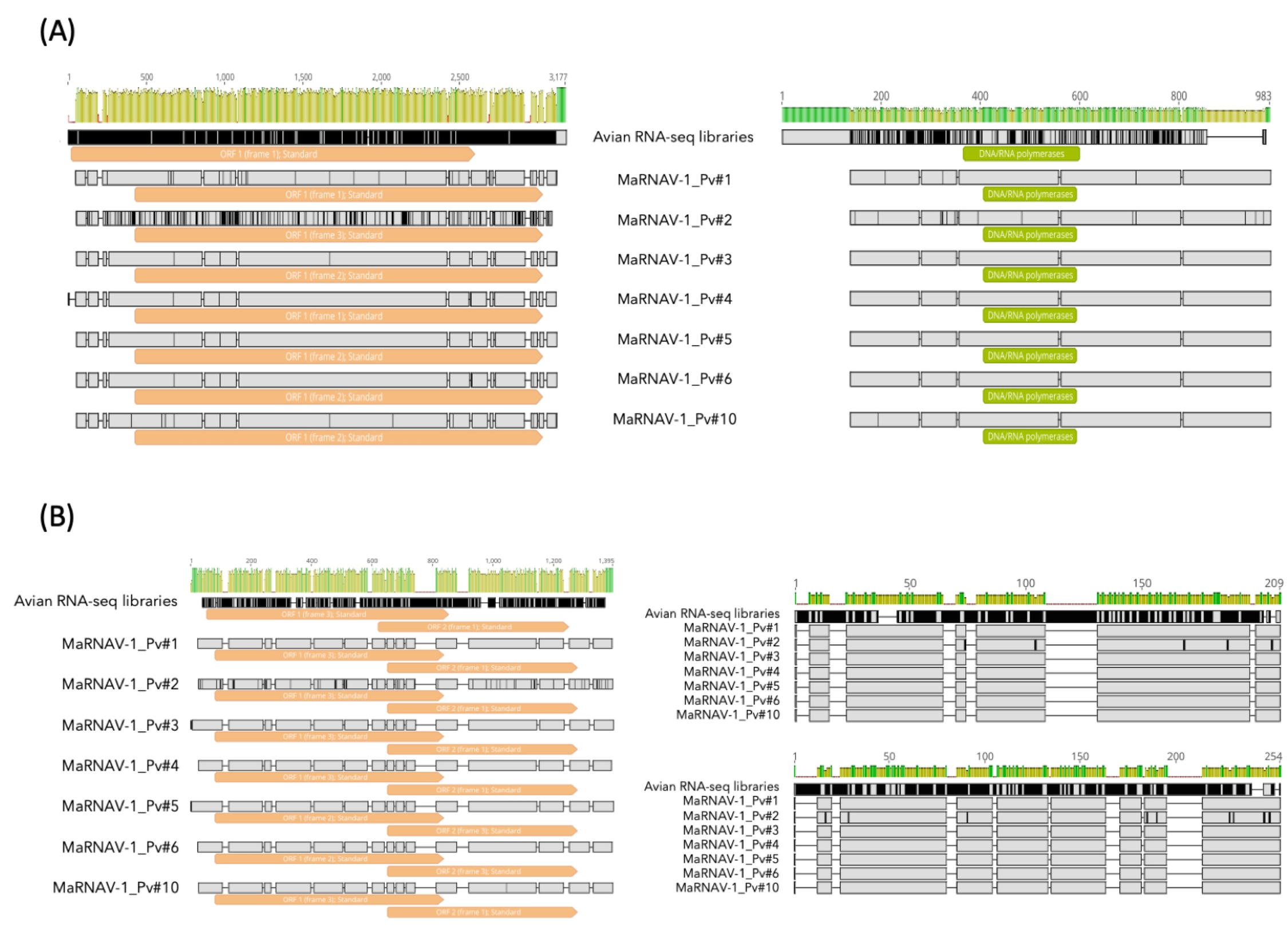

Supplement: S9 Fig — (A) Analysis of segment I. (B) Analysis of segment II. Nucleotide alignments are shown on the left and protein alignments are shown on the right (top—ORF1; bottom—ORF2). Orange boxes: predicted ORF using standard genetic code. Light green boxes: InterProScan domain prediction. Yellow to green plots: level of sequence conservation. (TIF) [file ppat.1008216.s017.tif]

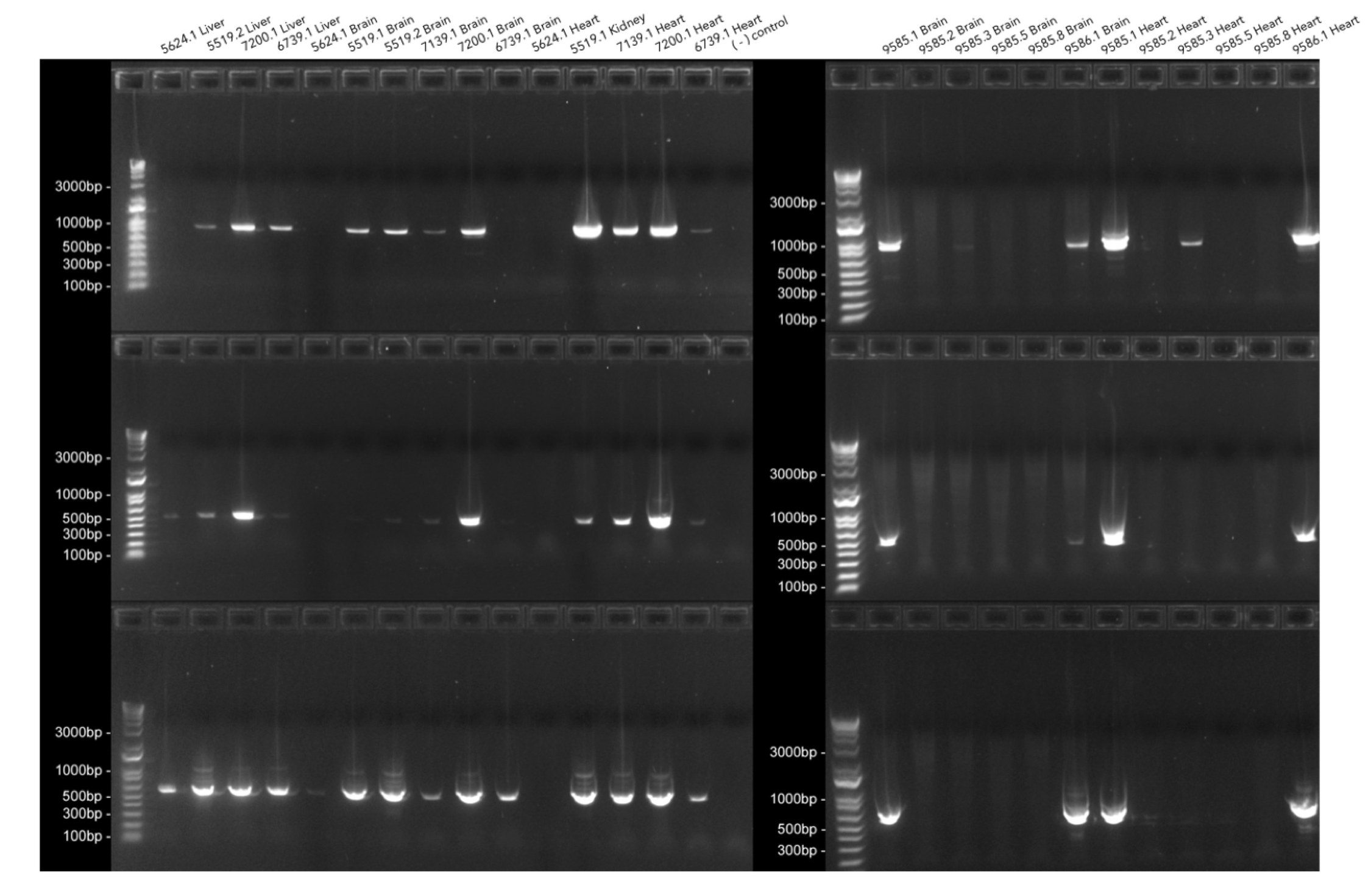

Supplement: S10 Fig — Top: Leucocytozoon CytB PCR; Middle: MaRNAV-2 segment I homolog detection; Bottom: MaRNAV-2 segment II homolog detection. (TIF) [file ppat.1008216.s018.tif]

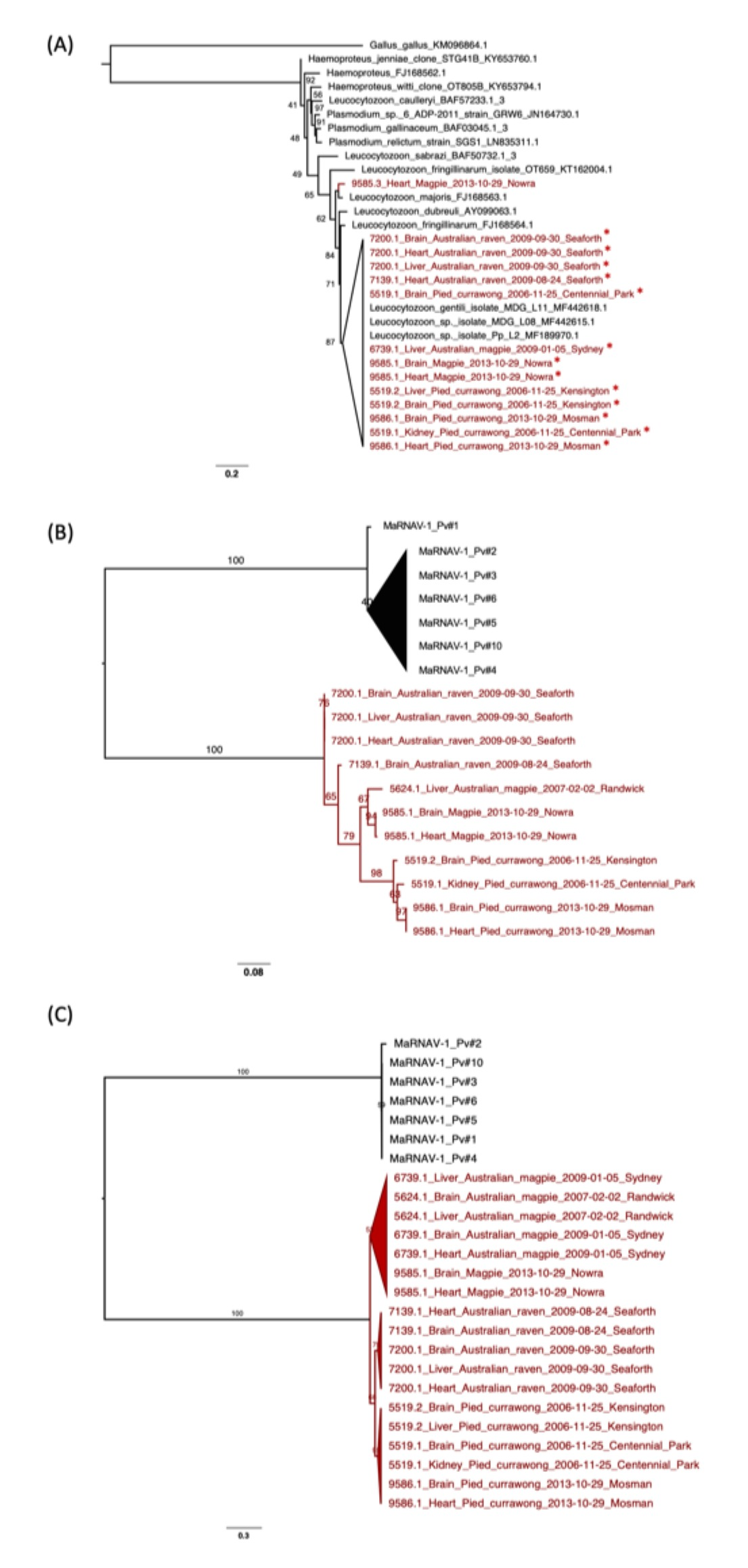

Supplement: S11 Fig — (A) Hematozoa CytB phylogeny. The CytB sequence from Gallus gallus is used as an outgroup. Samples positive for MaRNAV-2 l are marked with *. (B) MaRNAV-2 segment I phylogeny; (C) MaRNAV-2 segment II phylogeny. Sequences from bird samples are shown in red. (TIF) [file ppat.1008216.s019.tif]
