# Supplementary material for: Novel RNA viruses associated with Plasmodium vivax in human malaria and Leucocytozoon parasites in avian disease
Source: PLoS Pathog. 2019 Dec 30;15(12):e1008216. doi: 10.1371/journal.ppat.1008216 (PMC6953888; doi:10.1371/journal.ppat.1008216)
Supplement: S1 References — (DOCX) [file ppat.1008216.s020.docx]

**S1 Supporting references**

1. Roth A, Adapa SR, Zhang M, Liao X, Saxena V, Goffe R, et al. Unraveling the *Plasmodium vivax* sporozoite transcriptional journey from mosquito vector to human host. Sci Rep. 2018; 8: 12183. doi: 10.1038/s41598-018-30713-1.
2. Gural N, Mancio-Silva L, Miller AB, Galstian A, Butty VL, Levine SS, et al. *In vitro* culture, drug sensitivity, and transcriptome of *Plasmodium vivax* hypnozoites. Cell Host Microbe. 2018; 23: 395–406. doi: 10.1016/j.chom.2018.01.002.
3. Kim A, Popovici J, Vantaux A, Samreth R, Bin S, Kim S, et al. Characterization of *P. vivax* blood stage transcriptomes from field isolates reveals similarities among infections and complex gene isoforms. Sci Rep. 2017; 7: 7761. doi: 10.1038/s41598-017-07275-9.
4. Jex A, Mueller I, Kappe S, Mikolajcjak S, Sattabongkot J, Patrapuvich R, et al. Transcriptome and histone epigenome of *Plasmodium vivax* salivary-gland sporozoites point to tight regulatory control and potential mechanisms for liver-stage differentiation. bioRxiv. 2018; doi:10.1101/145250.
5. Rojas-Pena MC, Arafat DC, Velasquez JM, Garimalla SC, Arevalo-Herrera M, Herrera S, et al. Profiling gene expression of the host response to a *Plasmodium vivax* irradiated sporozoite immunization and infectious challenge. bioRxiv. 2018; doi:10.1101/501957.
6. Rojas-Peña ML, Vallejo A, Herrera S, Gibson G, Arévalo-Herrera M. Transcription profiling of malaria-naïve and semi-immune Colombian volunteers in a *Plasmodium vivax* sporozoite challenge. PLoS Negl Tropi Dis. 2015; 9: e0003978. doi:10.1371/journal.pntd.0003978.
7. Zhu L, Mok S, Imwong M, Jaidee A, Russell B, Nosten F, et al. New insights into the *Plasmodium vivax* transcriptome using RNA-Seq. Sci Rep. 2016; 6:20498. doi: 10.1038/srep20498.
8. Padley D, Moody AH, Chiodini PL, Saldanha J. Use of a rapid, single-round, multiplex PCR to detect malarial parasites and identify the species present. Ann Trop Med Parasitol. 2003; 97: 131-137. doi:10.1179/000349803125002977.
9. Imwong M, Tanomsing N, Pukrittayakamee S, Day NPJ, White NJ, Snounou G. Spurious amplification of a *Plasmodium vivax* small-subunit RNA gene by use of primers currently used to detect *P. knowlesi*. J Clin Micro. 2009; 47: 4173-4175. doi:10.1128/jcm.00811-09.
10. Singh B, Snounou G, Abdullah MS, Rahman HA, Bobogare A, Cox-Singh J. A genus- and species-specific nested polymerase chain reaction malaria detection assay for epidemiologic studies. Am J Trop Med Hyg. 1999; 60: 687-692. doi:10.4269/ajtmh.1999.60.687.
11. Pacheco MA, Cepeda AS, Bernotienė R, Lotta IA, Matta NE, Valkiūnas G, et al. Primers targeting mitochondrial genes of avian haemosporidians: PCR detection and differential DNA amplification of parasites belonging to different genera. Int J Parasitol. 2018; 48: 657–670. doi: 10.1016/j.ijpara.2018.02.003.
12. Quast C, Pruesse E, Yilmaz P, Gerken J, Schweer T, Yarza P, et al. The SILVA ribosomal RNA gene database project: improved data processing and web-based tools. Nuc Acids Res. 2013; 41: D590–596. doi: 10.1093/nar/gks1219.
13. Kopylova E, Noé L, Touzet H. SortMeRNA: fast and accurate filtering of ribosomal RNAs in metatranscriptomic data. Bioinformatics. 2012; 28: 3211–3217. doi: 10.1093/bioinformatics/bts611.
14. Kalvari I, Argasinska J, Quinones-Olvera N, Nawrocki EP, Rivas E, Eddy SR, et al. Rfam 13.0: shifting to a genome-centric resource for non-coding RNA families. Nuc Acids Res. 2018; 46: D335–D342. doi: 10.1093/nar/gkx1038.
15. Griffiths-Jones S. Annotating non-coding RNAs with Rfam. Curr Protocol Bioinformatics. 2005; Chapter 12: Unit 12.5. doi:10.1002/0471250953.bi1205s9.
16. Langmead B, Salzberg SL. Fast gapped-read alignment with Bowtie 2. Nat Meth. 2012; 9: 357–359. doi: 10.1038/nmeth.1923.
